# Supplementary material for: Lymphopenia and Early Variation of Lymphocytes to Predict In-Hospital Mortality and Severity in ED Patients with SARS-CoV-2 Infection
Source: J Clin Med. 2022 Mar 24;11(7):1803. doi: 10.3390/jcm11071803 (PMC8999889; doi:10.3390/jcm11071803)
Supplement: Supplementary file 1 [file jcm-11-01803-s001.zip › jcm-1634407-supplementary.pdf]

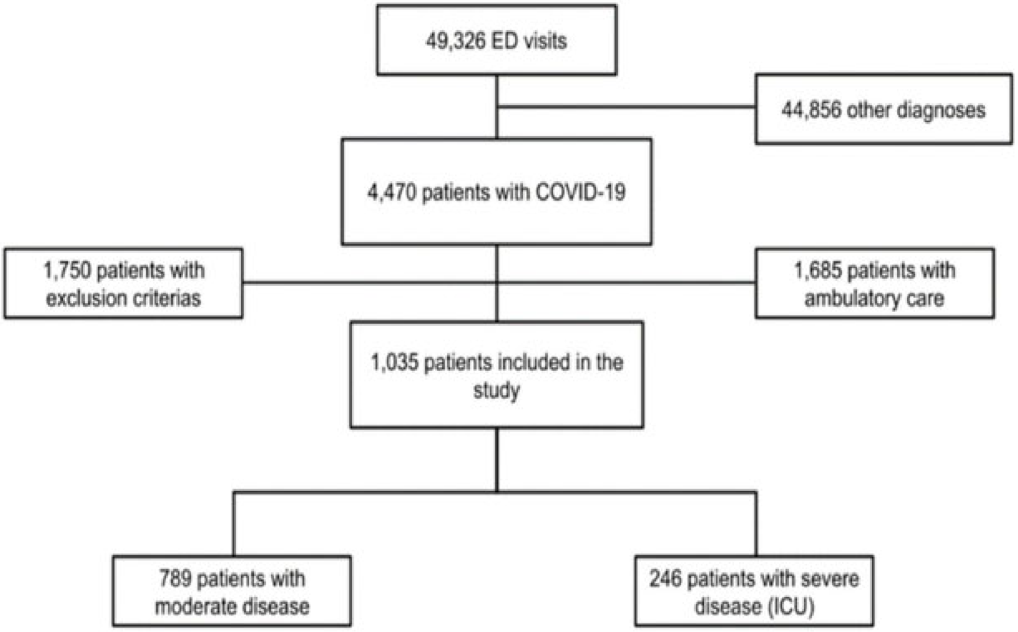


**Figure S1.** Flowchart of the study and procedure selection

**Table S1**. Prognostic value of lymphocytes and early variation of lymphocyte according age or gender to predict severity of disease

| Severity | Lymphocyte count | | Δ lymphocytes H24 | |
|---|---|---|---|---|
| | AUC [95% IC] | P | AUC [95% IC] | P |
| male | 55.7371 [ 50.6916 ; 60.7826 ] | 0.0244 | 62.4692 [ 57.134 ; 67.8045 ] | <0.001 |
| female | 55.2676 [ 47.7256 ; 62.8097 ] | 0.1644 | 58.933 [ 50.8892 ; 66.9768 ] | 0.0267 |
| AGE<58 | 64.6534 [ 56.5681 ; 72.7387 ] | <0.001 | 68.5554 [ 60.4667 ; 76.6441 ] | <0.001 |
| AGE=58,69 | 53.414 [ 45.7324 ; 61.0956 ] | 0.3832 | 60.1199 [ 52.0976 ; 68.1421 ] | 0.0139 |
| AGE=69,79 | 58.2317 [ 50.6485 ; 65.8148 ] | 0.0337 | 61.4056 [ 53.2386 ; 69.5726 ] | 0.0061 |

| | | | | |
|---|---|---|---|---|
| AGE≥79 | 63.5238 [ 50.334 ; 76.7137 ] | 0.039 | 52.9075 [ 39.4888 ; 66.3262 ] | 0.6588 |

**Table S2**. Prognostic value of lymphocytes and early variation of lymphocyte according age or gender to predict mortality

| Mortality | Lymphocyte count | | Δ lymphocytes H24 | |
|---|---|---|---|---|
| | AUC [95% IC] | P | AUC [95% IC] | P |
| male | 65.4739 [ 59.5053 ; 71.4424 ] | <0.001 | 56.3789 [ 49.5827 ; 63.1751 ] | 0.0656 |
| female | 55.6339 [ 46.2876 ; 64.9802 ] | 0.2332 | 63.7241 [ 54.8638 ; 72.5843 ] | 0.0022 |
| AGE<58 | 80.8926 [ 66.0277 ; 95.7575 ] | <0.001 | 51.4357 [ 37.2431 ; 65.6283 ] | 0.8362 |
| AGE=58,69 | 56.8512 [ 43.0295 ; 70.6729 ] | 0.3082 | 69.5767 [ 58.1491 ; 81.0044 ] | <0.001 |
| AGE=69,79 | 57.5609 [ 48.3711 ; 66.7507 ] | 0.1074 | 61.1524 [ 51.4125 ; 70.8924 ] | 0.023 |
| AGE≥79 | 56.5304 [ 48.1727 ; 64.8882 ] | 0.123 | 54.0022 [ 44.3554 ; 63.6489 ] | 0.4145 |
